# Towards novel BIOmarkers to diagnose SEPsis (BIOSEP) in the emergency room: a protocol for a multicentre, prospective cohort study

**DOI:** 10.1136/bmjopen-2025-103138

**Published:** 2025-08-01

**Authors:** Evelien Reijnders, Oren Turgman, Sebastiaan C M Joosten, Michiel Schinkel, Marleen A Slim, Renée A Douma, Hazra S Moeniralam, Hessel Peters-Sengers, Tom van der Poll, W Joost Wiersinga

**Affiliations:** 1 Center for Infection and Molecular Medicine, Amsterdam UMC Location AMC, Amsterdam, The Netherlands; 2 Department of Internal Medicine, Flevo Hospital, Almere, The Netherlands; 3 Department of Internal Medicine, Sint Antonius, Nieuwegein, The Netherlands; 4 Department of Epidemiology and Data Science, Amsterdam UMC Location VUmc, Amsterdam, The Netherlands; 5 Department of Internal Medicine, Division infectious Diseases, Amsterdam UMC Locatie AMC, Amsterdam, The Netherlands

**Keywords:** Sepsis, Emergency Service, Hospital, Shock, Septic, IMMUNOLOGY, INFECTIOUS DISEASES

## Abstract

**Abstract:**

**Introduction:**

The international Surviving Sepsis Campaign guidelines highlight the need for recognising sepsis in a timely and accurate manner. Early diagnosis and treatment of sepsis are crucial and associated with reduced mortality. In the early stages of sepsis, heterogeneous clinical signs are difficult to interpret, often leading to missed or delayed diagnoses, as well as inappropriate or late use of antibiotics. There is an urgent need to quickly and accurately identify patients with potential infection in the emergency department (ED) who are at risk of progressing along the infection-sepsis spectrum. This study aims to compare the immune responses of ED patients presenting with a (suspected) infection, both with and without sepsis, to gain insights into immune aberrations and identify novel biomarkers linked to an increased risk of developing sepsis and its sequelae.

**Methods and analysis:**

A prospective observational cohort study across three hospitals in the Netherlands will be conducted. Adults presenting to the ED of these hospitals, with a (suspected) infection and a Modified Early Warning Score of 2 or higher, will be eligible for enrolment. We aim to include up to 3330 patients. The main study parameters will be characterisation of the host immune and metabolic response using multiomics analysis, Raman spectroscopy, mass spectrometry and gut microbiota profiling in relation to clinical outcomes such as 30-day mortality, length of hospital stay, readmission and postsepsis sequelae.

**Ethics and dissemination:**

Verbal and written informed consent will be obtained from all participants or their legal representatives. The study was approved by the Amsterdam University Medical Centre Ethics Committee (No. 2022.0279) and will adhere to the Declaration of Helsinki and the Medical Research Involving Human Subjects Act (WMO). Research results will be published in peer-reviewed journals.

**Trail registration number:**

ClinicalTrials.gov, NCT06178822.

**STRENGTHS AND LIMITATIONS OF THIS STUDY**

- BIOmarkers to diagnose SEPsis is a multicentre emergency department (ED) study providing insight into the early immune response in infection and sepsis.
- Broad inclusion criteria capture a wide range of infections, enabling differentiation between rapid recovery and critical illness.
- Integration of advanced analyses, including multiomics and microbiome profiling, allows exploration of biological processes beyond conventional biomarkers.
- Deferred consent enables inclusion of severely ill patients unable to provide consent at time of ED admission.
- Wide-ranging inclusion criteria may result in a low number of severe cases or confirmed infections.

## Introduction

 Sepsis continues to account for nearly 20% of all deaths worldwide, despite a global reduction in disease burden,^1^ and remains a major contributor to in-hospital mortality.^2^ The Surviving Sepsis Campaign has highlighted diagnosis as a critical challenge.^3^ Early diagnosis and treatment are crucial, as they are associated with lower mortality rates.^4^ However, in routine clinical practice, there is often a significant delay after admission before acting on the suspicion of a severe infection, such as administering antibiotics.^5^ In the early stages of sepsis, the infection source may be unclear, and clinical signs may be indistinguishable from those of non-infectious diseases, resulting in missed or delayed diagnoses.^6^

For over 25 years, patients with an infection who met two or more systemic inflammatory response syndrome (SIRS) criteria were diagnosed with sepsis.^7^ However, due to the limited specificity of the SIRS criteria, sepsis was redefined in 2016 to focus on the presence of organ failure, as measured by the sequential organ failure assessment (SOFA) score, in patients with a (suspected) infection.^4^ Since the SOFA score requires laboratory testing, the quick SOFA (qSOFA) score was introduced for early sepsis recognition at the bedside, based on three parameters: systolic blood pressure, respiratory rate and Glasgow Coma Scale. Unfortunately, many clinical scores, including qSOFA and the Early Warning Score, lack sensitivity and prognostic value in predicting sepsis and mortality.^8 9^ The most recent Surviving Sepsis Campaign guidelines recommend using SIRS, the National Early Warning Score (NEWS) or the Modified Early Warning Score (MEWS) as sepsis screening tools,^3^ with MEWS and NEWS being the most used.^10^ To address the limitations of clinical scores, several biomarkers have been evaluated, yet none have demonstrated sufficient specificity or sensitivity for routine clinical use.^11^,^13^ In addition, classical microbiological cultures are time-consuming and often yield negative results, further highlighting the absence of an ideal diagnostic tool for sepsis.^14^ The host response in sepsis is heterogeneous, with distinct biological subgroups identified in intensive care unit (ICU) cohorts, suggesting that a single biomarker is unlikely to enable accurate diagnosis.^15 16^ To address this complexity, multiomics approaches are needed, yet such strategies have rarely been applied in the emergency department (ED), particularly in the early stages of sepsis.^11^

Data from the USA indicate that sepsis is a common presentation in the ED and accounts for roughly one-third of all hospital admissions that result in death.^17^ Even after hospital discharge, patients initially admitted for sepsis continue to have an increased risk of death.^18^ US data showed that one-third of sepsis survivors die within the following year, with half of these deaths related to sepsis complications. Additionally, one-sixth of sepsis survivors experience severe, persistent physical disability or cognitive impairment.^19^ Consequently, the Surviving Sepsis Campaign guidelines now recommend assessing and observing the physical, cognitive and emotional health of patients after hospital discharge for sepsis.^3^ Although the challenges of critical illness survivorship are increasingly documented, there are still relatively few studies on enhancing recovery and identifying patients at increased risk for postsepsis health problems.^18^

There is a pressing need to rapidly and effectively identify ED patients at risk of progressing along the infection-sepsis spectrum. The objectives of the present study are (1) to compare the immune response of patients with and without sepsis presenting to the ED with (suspected) infection, (2) to identify immune response aberrations and biomarkers associated with an increased risk of developing sepsis in patients presenting to the ED with (suspected) infection and (3) to determine the long-term cognitive and physical sequelae of sepsis after hospital discharge.

We will investigate the immune response using transcriptomic, metabolomic and proteomic data to gain insights into sepsis pathophysiology and to develop methods for stratification of patients along the infection-to-sepsis spectrum.

## Methods and analysis

### Study design and settings

This is a multicentre, prospective, observational cohort study conducted across three hospitals in the Netherlands: Amsterdam University Medical Centre and two peripheral hospitals, the Flevo Hospital in Almere and St. Antonius Hospital in Nieuwegein.

### Study population and recruitment

Adult (≥18 years) patients presenting to the ED with suspected or confirmed infection and a MEWS of ≥2 will be screened for eligibility. Components of the MEWS can be found in online supplemental table 1. Lack of informed consent by patients or their legal representative is the only criterion for exclusion. Potential participants will be approached for written informed consent. If a patient is unable or incapable of providing informed consent during the screening process, consent will be sought from the patient’s legal representative. Once the patient is no longer incapacitated, written informed consent will be obtained from the patient.

### Study procedures and follow-up

**Figure 1:**
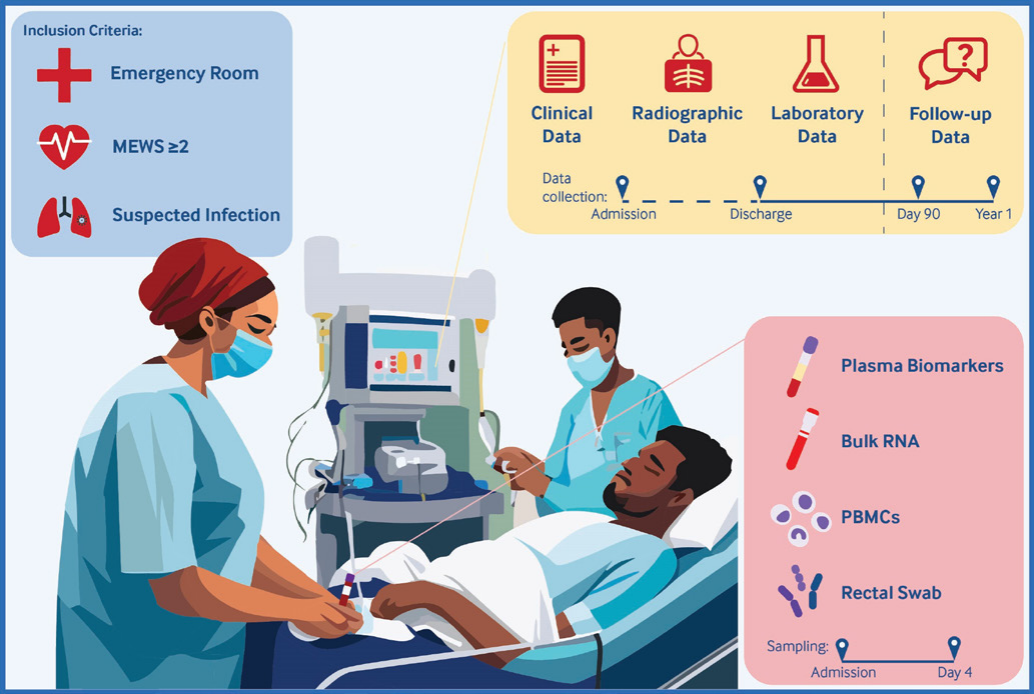
Graphical abstract of the BIOmarkers to diagnose SEPsis (BIOSEP) study. Overview of the study design illustrating the inclusion criteria and the nature and timeline of sample and data collection. MEWS, Modified Early Warning Score; PBMCs, peripheral blood mononuclear cells.

Samples will be collected in the ED at baseline and, if the patient remains hospitalised, on day 4 (range from 3 to 5). Questionnaires will be administered at 90 days and 1 year following the ED presentation. A summary of the study procedures and sample collection is provided in figure 1. Baseline and clinical data will be collected from the patient’s medical chart until the day of discharge or death. A detailed timeline of the clinical data collected throughout the study is outlined in table 1.

**Table 1:** Data collection timeline

Collected samples and data	Day 0	During admission	3 months	1 year
Plasma, PBMCs, PAXgene samples	X	Day 4		
Rectal swab	X			
Demographics and medical history	X			
Vital parameters	X	Day 4		
Laboratory values	X	Day 4		
Administered medication		X		
Radiology results		X		
Microbiology test		X		
Physician diagnosis	X	Discharge		
Adjudicated diagnoses			X	
Adjudicated complications			X	
Readmission			X	X
Mortality		X	X	X
Postsepsis sequelae			X	X

PBMCs, peripheral blood mononuclear cells.

#### Blood sampling and rectal swabs

Blood samples will be collected using ethylenediaminetetraacetic acid (EDTA) tubes for plasma extraction, heparin tubes for isolation of peripheral blood mononuclear cells (PBMCs) and single-cell proteomics using Smart Tubes (Las Vegas, NV, USA) and PAXgene RNA tubes for RNA extraction. The maximum extracted volume of blood will be 67.5 mL at baseline and 57.5 mL at the follow-up visit. Plasma and PAXgene RNA samples will be stored at −70°C and PBMCs at −196°C (liquid nitrogen) in the Amsterdam UMC Biobank.

Rectal swabs (FLOQSwabs 552C, Copan, CA, US) will be collected on day 0 and used for gut microbiota analysis. Rectal swabs are commonly used in clinical practice and are easier to obtain at a specific time point compared with faecal samples.^20^ They have been shown to accurately represent faecal microbiota composition and functionality in both critically ill patients and healthy controls.^21 22^ The swabs are inserted approximately 3 cm into the anal canal by a research team member, healthcare provider or the participant themselves. The swabs will be placed in a container with 500 µL of reduced transport fluid (buffer) and may be kept at room temperature for up to 2 hours before being stored at −70°C.

#### Questionnaires

Approximately 90 days (±2 weeks) and 1 year (±1 month) after presentation to the ED, short questionnaires will be administered. The questionnaires incorporate questions from two validated questionnaires and address mortality, readmission, healthcare use and the sequelae of sepsis.^23 24^ This approach allows us to systematically evaluate the long-term effects of sepsis. The questionnaires are provided in online supplemental material 1.

#### Measurement of immune profiles

The immune response analyses used include mass spectrometry, proteomics, transcriptomics, metabolomics, microbiomics and Raman spectroscopy.

Raman spectroscopy is a non-destructive analytical technique that uses inelastic scattering of light to provide information on chemical composition of samples.^25 26^ Raman spectroscopy will be applied to plasma samples. In this way, it may be able to recognise immunological profiles and identify individuals at high risk of developing sepsis. Host response biomarkers, including those related to coagulation, inflammation and endothelial activation, will be measured in EDTA plasma using Luminex technology. In addition, a proteomic assessment of a broad range of mediators will be conducted using Olink technology (Olink Proteomics, Uppsala, Sweden). Targeted proteomic analysis using Olink technology has been used to identify pathophysiologically relevant alterations in proteins among critically ill sepsis patients.^27^ Furthermore, single-cell proteomics analyses will be performed using mass cytometry, focusing on cell signalling in innate immunity. Gene transcription profiles will be assessed through both whole-blood and single-cell RNA sequencing in a targeted subgroup of patients, using PAXgene tubes and viably frozen PBMCs (Becton-Dickinson, Breda, Netherlands). Identifying transcriptomic endotypes in sepsis patients can provide valuable information about the prognosis of sepsis patients receiving critical care.^15^ A mass spectrometry-based metabolomics platform will be used to assess metabolic profiles in patient plasma. Liquid chromatography-mass spectrometry-based analysis offers opportunity to explore potential sepsis biomarkers.^28^ Microbiota sequencing will be performed. In short, a repeated bead-beating method was used for DNA extraction. 16S ribosomal RNA gene sequencing will be performed on an Illumina system (San Diego, CA, USA) and further preprocessing of sequence data as described.^29 30^

### Current status and protocol changes

Recruitment began on 25 October 2022 and is expected to conclude in 2028. Since the start of recruitment, two amendments have been made to this protocol. In the first amendment, the following changes were made and approved by the METC:

- The first follow-up date was changed from day 7 to day 4 and the second follow-up date from day 60 to day 90.
- The possibility of deferred consent was included in the study for temporarily incapacitated patients.
- A subgroup of patients for which faeces samples will be collected was defined.
- The Flevo Hospital was added as an inclusion centre.

In the second amendment, the following changes were made and approved by the METC:

- Race and ethnicity were explicitly added as demographic variables to be collected.
- Follow-up questionnaires were made available for digital use by included patients.
- The St. Antonius hospital was added as an inclusion centre.

The current version of the original study protocol can be found in online supplemental material 2.

### Study endpoints

The main study parameters will be characterisation of the host immune and metabolic response using multiomics analysis, Raman spectroscopy, mass spectrometry and gut microbiota profiling in relation to clinical outcomes such as 30-day mortality, length of hospital stay, readmission and postsepsis sequelae. The main study parameters and other relevant study variables that will be collected are detailed in table 2.

**Table 2:** Overview of recorded outcomes

Main study parameters	
Host response	Multiomics, Raman spectroscopy, mass spectrometry, gut microbiota analysis
Disease severity	MEWS and SOFA scores
Infection state	Infection diagnosis including causative pathogens and sepsis diagnosis
Mortality rates	In-hospital, 30-day, 90-day and 1-year mortality
Length of stay	Duration of hospital and ICU stay
Readmission	All-cause readmission in the first year after ED presentation
Postsepsis sequelae	Sequelae up to 90 days postadmission

Other relevant study variables	
General demographics	Sex, age, height, weight, race, ethnicity
Medical history	Comorbidities, tobacco, alcohol and drug use, medication use prior to admission
Information prior to ED	Recent surgery, duration of symptoms and antibiotic exposure 3 months prior to ED
Vital signs	Vital signs measured for standard care
Laboratory values	Blood tests conducted as part of standard care
Microbiology tests	Positive cultures, PCR, serology and urine rapid tests for causative microorganisms
Radiology results	Radiology findings positive for infection
Administered medication	Antimicrobials, immunosuppressives, vasopressors and anticoagulants
Organ support	Mechanical ventilation, renal replacement therapy, ECMO, vasopressors
Physician diagnosis	Diagnosis on ED and discharge diagnosis
Complications	AKI, secondary infection, ARDS, myocardial infarction, delirium

* See questionnaires in online supplemental material 1.

AKI, acute kidney injury; ARDS, acute respiratory distress syndrome; ECMO, extracorporeal membrane oxygenation; ED, emergency department; ICU, intensive care unit; MEWS, Modified Early Warning Score; SOFA, sequential organ failure assessment.

### Sepsis and infection definitions

#### Infection diagnosis

In 2013, Klouwenberg *et al* developed and validated a handbook for post hoc adjudication of infection diagnoses in the ICU, which was based on the Centers for Disease Control and Prevention (CDC) surveillance definitions for specific types of infections.^31^ We have updated this handbook using the 2023 CDC surveillance definitions for types of infections.^32^ Additionally, we have expanded it to include common complications of infections and sepsis, including delirium, secondary infections, acute respiratory distress syndrome, acute kidney injury, myocardial infarction, stroke, deep venous thrombosis and pulmonary embolism, based on the most recent versions of consensus definitions.^33^,^38^ Since gold standards are lacking for many infectious diagnoses, it is imperative to use an adjudication system to determine presence and source of infections reliably.^39^

Each patient will be independently reviewed by two experienced physicians, who will use all available data from the medical chart during the admission period. The plausibility of the infection (rated as none, possible, probable or definite), the most likely causative pathogen and sepsis-related complications during the infection period will be assessed based on the diagnostic handbook. In the event of a disagreement between the two reviewers, a third reviewer will evaluate the case independently. If the third reviewer still disagrees with both previous reviewers, a final diagnosis will be made by an independent adjudication committee consisting of specialists in internal medicine. The use of the updated diagnostic handbook in the ED and the interobserver agreement will be evaluated in a separate validation study.

#### Sepsis diagnosis

Sepsis will be defined according to the 2016 Third International Consensus Definitions for Sepsis and Septic Shock (Sepsis-3)^4^ as life-threatening organ dysfunction caused by a dysregulated host response to infection. Organ dysfunction is measured by a change in the SOFA score of ≥2. In cases where prior components of the SOFA score are unavailable, a baseline score of ‘0’ will be assumed. Assessing the pulmonary component can be challenging, since calculating the PaO2/FiO2 ratio requires arterial blood gases, which are not routinely available in the ED. In such cases, we will use a modified SOFA (mSOFA) score which uses the SpO2/FiO2 (SF) ratio as an alternative for assessing respiratory dysfunction. Cut-off values for SF ratios have been validated in large datasets on the ED, ICU and ward.^40^,^43^ Components of the mSOFA score and cut-off values can be found in online supplemental table 2. Sepsis will be adjudicated concurrently with infection diagnoses using the same independent review process.

### Sample size

A pilot survey (unpublished data) conducted by the Amsterdam University Medical Centre team across both EDs over a 60-day period (weekdays from November 2019 to February 2020) identified 279 patients with a suspected infection and MEWS of ≥3, of whom 30 (11%) were diagnosed with sepsis. Extrapolating these findings suggests that approximately 1674 ED visits annually will involve patients with a suspected infection and MEWS of ≥3, of whom approximately 180 will be diagnosed with sepsis. The survey also showed that the overall ED population with MEWS ≥2 is twice as large. Using this lower MEWS threshold increases the chances of identifying patients at risk of developing sepsis. With this strategy, we aim to enrol 3300 participants in total.

### Statistical analysis

Data will be summarised using means and SD, or medians and interquartile ranges, depending on distribution of the data. Comparisons between groups will use appropriate parametric or non-parametric tests, depending on data distribution. Missing data will be addressed using multiple imputation analysis, if missing at random. Linear mixed effect models will account for dependencies in repeated measures. Dimensionality reduction techniques, such as principal component analysis and partial least squares discriminant analysis, will be used to explore and differentiate immunologic profiles within large transcriptomic or proteomic datasets. Regression models will be used to investigate associations between biomarkers and relevant outcomes. P values of <0.05 will be considered significant.

### Data management

All clinical data and outcomes will be manually extracted from the electronic health record into the Castor electronic data capture (Castor EDC) system. The questionnaires will be administered either by phone or digitally through Castor EDC. All patient data will be stored in accordance with local regulations and with the patient’s consent. Patient data will be pseudonymised to protect patient privacy. The encryption key will be available only to coordinating researchers. The research file will be password-protected and stored for 15 years. Inclusion in the BIOmarkers to diagnose SEPsis (BIOSEP) biobank means sample storage for up to 15 years. The study team will be responsible for data management. Local independent monitors will perform data validation checks and ensure that all study procedures comply with local regulations and protocol.

### Safety, adverse events and monitoring

The study will be monitored by the local clinical monitoring centre. According to the applicable Nederlandse Federatie van Universitair Medische Centra guidelines,^44^ the risk and burden to patients in this study are negligible. Given the negligible burden and risks for patients in this observational study, we will only report adverse events (AEs) and serious adverse events (SAEs) related to blood sampling. However, we do not expect any AEs or SAEs resulting from blood sampling. The investigator will report all SAEs to the sponsor promptly after becoming aware of them. The sponsor will then report these to the accredited Medical Ethical Committee that approved the protocol. For SAEs resulting in death or those that are life-threatening, reporting will occur within 7 days of first knowledge, with an additional 8 days allowed to complete the initial report. All other SAEs will be reported within 15 days of the sponsor’s first knowledge of the events.

### Patient and public involvement

None.

## Ethics and dissemination

The study was approved by the Amsterdam University Medical Centre’s Ethics Committee (No. 2022.0279) and will adhere to the Declaration of Helsinki and the Medical Research Involving Human Subjects Act (WMO). Verbal and written informed consent will be obtained from all participants or their legal representatives. Included patients were able to withdraw from the study at any time. Research results will be published in peer-reviewed journals.

## Discussion

The BIOSEP study is a large-scale investigation aimed at identifying early biomarkers relevant for the initial biological responses to infection and sepsis in an ED setting using a multiomics approach. Sepsis is a complex heterogeneous syndrome, involving an overactive inflammatory response alongside immune suppression, coagulation disruptions and alterations in the microbiome, all contributing to a failure to restore homeostasis.^45^ This heterogeneity suggests the existence of distinct biological phenotypes, underscoring the need to define the mechanisms of immune dysfunction to identify predictive markers and advance precision therapy.

Research into sepsis mechanisms has largely relied on animal models and data from ICU patients with sepsis, as defined by Sepsis-3 criteria.^4^ These studies have provided valuable insights into the pathophysiology, identifying endotypes based on gene expression profiles and immune responses.^15 16 46^ However, these findings primarily reflect advanced stages of the disease, by which point the window for early treatment has often passed. Timely detection and intervention are crucial for improving patient outcomes, yet our understanding of the initial immune responses remains insufficient.

A number of studies have applied omics-based approaches in ED settings, with promising results. For example, the transcriptomic classifiers IMX-BVN-2 and IMX-SEV-2, based on a 29-gene host expression signature, were able to differentiate between bacterial, viral and non-infectious illness (area under the curve (AUC) 0.80–0.90) and predict in-hospital mortality (AUC 0.84) among ED patients.^47 48^ A targeted proteomic study in paediatric ED patients used a three-protein panel (TRAIL, IP-10 and C-reactive protein) to distinguish bacterial from viral infections with high accuracy (AUC 0.90–0.92).^49^ Metabolomic profiling of blood samples from adult ED patients with suspected sepsis identified a six-metabolite panel that distinguished bacteremic sepsis from other acute conditions (AUC 0.93, accuracy 88%).^50^ While these studies support the potential of host-response-based diagnostics, they each focus on a single biological layer and typically address pathogen classification or compare highly selected patient groups. To date, no study has applied an integrative omics approach in a heterogeneous ED population of patients with suspected infection to enable early sepsis recognition and biologically meaningful patient stratification. The BIOSEP study aims to address this gap by focusing on ED patients with infections, at risk of progressing to sepsis. By employing a multiomics approach, BIOSEP seeks to unravel the host response during the early stages of sepsis and to identify new predictive biomarkers and potential therapeutic targets. This approach could enhance prognostic enrichment by linking early host response immune profiles to patient outcomes.

A key strength of our study is that we include a broad spectrum of ED patients with a suspected infection, capturing a range of patients with mild infections to severe sepsis. This design allows for effective discrimination between patients who become critically ill and those who recover rapidly. Longitudinal samples further provide insight into transient physiological immune activation versus pathological dysregulation of the immune system. The integration of diverse analytical approaches, including multiomics and microbiome profiling, allows for a comprehensive view of biological processes beyond conventional biomarkers like C-reactive protein and procalcitonin, whose predictive values for sepsis outcomes are limited.^11 51^ Another important strength is our ability to include severely ill patients unable to provide consent (eg, due to delirium), through consent from their legal representatives, ensuring representation of this vulnerable group.

However, the BIOSEP study faces several possible challenges. While broad inclusion criteria capture a diverse patient population, they may also result in a low number of events, such as sepsis or death. Since suspected infections are also included, not all patients are expected to have confirmed infections. Acute care research also presents logistical hurdles, leading to missed potential inclusions outside regular working hours. Enrolling patients in the acute phase can be particularly challenging, as immediate clinical care takes precedence, and patients may struggle to consider participation under their conditions. Additionally, while multiomics approaches provide rich datasets, translating these findings into practical bedside biomarkers remains a challenge.

Given its design, BIOSEP is primarily intended as a hypothesis-generating study. The large-scale integration of multiple omics data will allow for the identification of novel biomarker signatures and immune profiles associated with clinical outcomes. While external validation is beyond the scope of this study, the inclusion of patients from three diverse hospital settings may allow for internal cross-validation or subgroup analyses, enhancing the robustness and generalisability of the findings. These results may inform future validation studies in independent cohorts and guide the development of targeted diagnostic or therapeutic strategies.

To conclude, this multicentre study and biobank will provide critical insights into the immune dysregulation that occurs along the infection-sepsis spectrum and may offer new avenues for early prediction, diagnosis and treatment strategies.

## Supplementary material

10.1136/bmjopen-2025-103138 online supplemental file 1

## References

[1] Rudd KE, Johnson SC, Agesa KM (2020). Global, regional, and national sepsis incidence and mortality, 1990-2017: analysis for the Global Burden of Disease Study. Lancet.

[2] Liu V, Escobar GJ, Greene JD (2014). Hospital deaths in patients with sepsis from 2 independent cohorts. JAMA.

[3] Evans L, Rhodes A, Alhazzani W (2021). Surviving sepsis campaign: international guidelines for management of sepsis and septic shock 2021. Intensive Care Med.

[4] Singer M, Deutschman CS, Seymour CW (2016). The Third International Consensus Definitions for Sepsis and Septic Shock (Sepsis-3). JAMA.

[5] Seymour CW, Liu VX, Iwashyna TJ (2016). Assessment of Clinical Criteria for Sepsis: For the Third International Consensus Definitions for Sepsis and Septic Shock (Sepsis-3). JAMA.

[6] Klein Klouwenberg PMC, Cremer OL, van Vught LA (2015). Likelihood of infection in patients with presumed sepsis at the time of intensive care unit admission: a cohort study. Crit Care.

[7] Bone RC, Balk RA, Cerra FB (1992). Definitions for Sepsis and Organ Failure and Guidelines for the Use of Innovative Therapies in Sepsis. *Chest*.

[8] Liu Y-C, Luo Y-Y, Zhang X (2019). Quick Sequential Organ Failure Assessment as a prognostic factor for infected patients outside the intensive care unit: a systematic review and meta-analysis. Intern Emerg Med.

[9] Hamilton F, Arnold D, Baird A (2018). Early Warning Scores do not accurately predict mortality in sepsis: A meta-analysis and systematic review of the literature. J Infect.

[10] Azijli K, Minderhoud T, Mohammadi P (2021). A prospective, observational study of the performance of MEWS, NEWS, SIRS and qSOFA for early risk stratification for adverse outcomes in patients with suspected infections at the emergency department. Acute Med.

[11] Turgman O, Schinkel M, Wiersinga WJ (2023). Host Response Biomarkers for Sepsis in the Emergency Room. Crit Care.

[12] van Engelen TSR, Wiersinga WJ, Scicluna BP (2018). Biomarkers in Sepsis. Crit Care Clin.

[13] Pierrakos C, Velissaris D, Bisdorff M (2020). Biomarkers of sepsis: time for a reappraisal. Crit Care.

[14] Rothman M, Levy M, Dellinger RP (2017). Sepsis as 2 problems: Identifying sepsis at admission and predicting onset in the hospital using an electronic medical record-based acuity score. J Crit Care.

[15] Sinha P, Kerchberger VE, Willmore A (2023). Identifying molecular phenotypes in sepsis: an analysis of two prospective observational cohorts and secondary analysis of two randomised controlled trials. Lancet Respir Med.

[16] Scicluna BP, van Vught LA, Zwinderman AH (2017). Classification of patients with sepsis according to blood genomic endotype: a prospective cohort study. Lancet Respir Med.

[17] Rhee C, Dantes R, Epstein L (2017). Incidence and Trends of Sepsis in US Hospitals Using Clinical vs Claims Data, 2009-2014. JAMA.

[18] Prescott HC, Sussman JB, Wiersinga WJ (2020). Postcritical illness vulnerability. Curr Opin Crit Care.

[19] Prescott HC, Angus DC (2018). Enhancing Recovery From Sepsis: A Review. JAMA.

[20] Radhakrishnan ST, Gallagher KI, Mullish BH (2023). Rectal swabs as a viable alternative to faecal sampling for the analysis of gut microbiota functionality and composition. Sci Rep.

[21] Bansal S, Nguyen JP, Leligdowicz A (2018). Rectal and Naris Swabs: Practical and Informative Samples for Analyzing the Microbiota of Critically Ill Patients. mSphere.

[22] Budding AE, Grasman ME, Eck A (2014). Rectal swabs for analysis of the intestinal microbiota. PLoS One.

[23] Huang CY, Daniels R, Lembo A (2019). Life after sepsis: an international survey of survivors to understand the post-sepsis syndrome. Int J Qual Health Care.

[24] Chopra V, Flanders SA, O’Malley M (2021). Sixty-Day Outcomes Among Patients Hospitalized With COVID-19. Ann Intern Med.

[25] Neugebauer U, Trenkmann S, Bocklitz T (2014). Fast differentiation of SIRS and sepsis from blood plasma of ICU patients using Raman spectroscopy. J Biophotonics.

[26] Arend N, Pittner A, Ramoji A (2020). Detection and Differentiation of Bacterial and Fungal Infection of Neutrophils from Peripheral Blood Using Raman Spectroscopy. Anal Chem.

[27] Van Nynatten LR, Slessarev M, Martin CM (2022). Novel plasma protein biomarkers from critically ill sepsis patients. Clin Proteomics.

[28] Li Y, Wang C, Chen M (2023). Metabolomics-based study of potential biomarkers of sepsis. Sci Rep.

[29] Haak BW, Brands X, Davids M (2022). Bacterial and Viral Respiratory Tract Microbiota and Host Characteristics in Adults With Lower Respiratory Tract Infections: A Case-Control Study. Clin Infect Dis.

[30] Kullberg RFJ, Wikki I, Haak BW (2024). Association between butyrate-producing gut bacteria and the risk of infectious disease hospitalisation: results from two observational, population-based microbiome studies. Lancet Microbe.

[31] Klein Klouwenberg PMC, Ong DSY, Bos LDJ (2013). Interobserver agreement of Centers for Disease Control and Prevention criteria for classifying infections in critically ill patients. Crit Care Med.

[32] Promotion. NCfEaZIDUSDoHQ (2023). CDC/NHSN surveillance definitions for specific types of infections. https://stacks.cdc.gov/view/cdc/127235.

[33] American Psychiatric Association (2013). Diagnostic and statistical manual of mental disorders.

[34] Thygesen K, Alpert JS, Jaffe AS (2018). Fourth Universal Definition of Myocardial Infarction (2018). Circulation.

[35] Matthay MA, Arabi Y, Arroliga AC (2024). A New Global Definition of Acute Respiratory Distress Syndrome. Am J Respir Crit Care Med.

[36] Sacco RL, Kasner SE, Broderick JP (2013). An updated definition of stroke for the 21st century: a statement for healthcare professionals from the American Heart Association/American Stroke Association. Stroke.

[37] Bellomo R, Ronco C, Kellum JA (2004). Acute renal failure - definition, outcome measures, animal models, fluid therapy and information technology needs.The Second International Consensus Conference of the Acute Dialysis Quality Initiative (ADQI) Group. Crit Care.

[38] Rivera-Lebron B, McDaniel M, Ahrar K (2019). Diagnosis, Treatment and Follow Up of Acute Pulmonary Embolism: Consensus Practice from the PERT Consortium. Clin Appl Thromb Hemost.

[39] Whitfield NN, Hogan CA, Chenoweth J (2024). A standardized protocol using clinical adjudication to define true infection status in patients presenting to the emergency department with suspected infections and/or sepsis. Diagn Microbiol Infect Dis.

[40] Pandharipande PP, Shintani AK, Hagerman HE (2009). Derivation and validation of Spo2/Fio2 ratio to impute for Pao2/Fio2 ratio in the respiratory component of the Sequential Organ Failure Assessment score. Crit Care Med.

[41] Rice TW, Wheeler AP, Bernard GR (2007). Comparison of the SpO2/FIO2 ratio and the PaO2/FIO2 ratio in patients with acute lung injury or ARDS. Chest.

[42] Rahmatinejad Z, Reihani H, Tohidinezhad F (2019). Predictive performance of the SOFA and mSOFA scoring systems for predicting in-hospital mortality in the emergency department. Am J Emerg Med.

[43] Grissom CK, Brown SM, Kuttler KG (2010). A modified sequential organ failure assessment score for critical care triage. Disaster Med Public Health Prep.

[44] NFU (2023). Richtlijn kwaliteitsborging mensgebonden onderzoek. https://www.nfu.nl/sites/default/files/2023-09/23.01358_Richtlijn_Kwaliteitsborging_Mensgebonden_Onderzoek_2023.pdf.

[45] van der Poll T, van de Veerdonk FL, Scicluna BP (2017). The immunopathology of sepsis and potential therapeutic targets. Nat Rev Immunol.

[46] Davenport EE, Burnham KL, Radhakrishnan J (2016). Genomic landscape of the individual host response and outcomes in sepsis: a prospective cohort study. Lancet Respir Med.

[47] Galtung N, Diehl-Wiesenecker E, Lehmann D (2022). Prospective validation of a transcriptomic severity classifier among patients with suspected acute infection and sepsis in the emergency department. Eur J Emerg Med.

[48] Mayhew MB, Buturovic L, Luethy R (2020). A generalizable 29-mRNA neural-network classifier for acute bacterial and viral infections. Nat Commun.

[49] van Houten CB, de Groot JAH, Klein A (2017). A host-protein based assay to differentiate between bacterial and viral infections in preschool children (OPPORTUNITY): a double-blind, multicentre, validation study. Lancet Infect Dis.

[50] Kauppi AM, Edin A, Ziegler I (2016). Metabolites in Blood for Prediction of Bacteremic Sepsis in the Emergency Room. PLoS One.

[51] Schuurman AR, Reijnders TDY, Kullberg RFJ (2021). Sepsis: deriving biological meaning and clinical applications from high-dimensional data. Intensive Care Med Exp.

