# Supplementary material for: Towards novel BIOmarkers to diagnose SEPsis (BIOSEP) in the emergency room: a protocol for a multicentre, prospective cohort study
Source: BMJ Open. 2025 Aug 1;15(7):e103138. doi: 10.1136/bmjopen-2025-103138 (PMC12314967; doi:10.1136/bmjopen-2025-103138)
Supplement: online supplemental file 1 [file bmjopen-15-7-s001.docx]

**Supplementary file**

**TABLE OF CONTENTS**

Supplementary table 1. Modified Early Warning Score (MEWS)

Supplementary table 2. Modified Sequential Organ Failure Assessment (SOFA) score

Supplementary material 1. Questionnaires day 90 and 1 year

Supplementary material 2. Original protocol

**Supplementary table 1. Modified Early Warning Score (MEWS)**

| Supplementary table 1. Modified Early Warning Score | | | | | | | | |
| --- | --- | --- | --- | --- | --- | --- | --- | --- |
| Score | **3** | **2** | **1** | **0** | | **1** | **2** | **3** |
| Respiratory rate (breaths/min) |  | <9 |  | 9-14 | | 15-20 | 21-30 | >30 |
| Saturation with therapy (%) | <90 |  |  |  | |  |  |  |
| Heartrate (beats/min) |  | <40 | 40-50 | 51-100 | | 101-110 | 111-130 | >130 |
| Systolic blood pressure (mmHg) | <70 | 70-80 | 81-100 | 101-200 | |  | >200 |  |
| Urine production (mL) |  |  | <75mL in the last 4 hours |  | |  |  |  |
| Consciousness |  |  |  | Alert | | Response to addressing | Response to pain | Unresponsive |
| Temperature (°C) |  | <35.1 | 35.1-36.5 | 36.6-37.5 | | >37.5 |  |  |
| Worries |  |  | If you are worried about the patient’s condition |  | |  |  |  |
|  | | | | |  | | | |

**Supplementary table 2. Modified Sequential Organ Failure Assessment (SOFA) score**

| Supplementary table 2. Modified Sequential Organ Failure Assessment (SOFA) score | |
| --- | --- |
| Variables | **Points** |
| SpO2/FiO2, mmHg |  |
| >400 | 0 |
| 315-400 | +1 |
| 235-315 | +2 |
| 150-235 | +3 |
| <150 | +4 |
| Platelets, x10^3^/μL | |
| >150 | 0 |
| 100-149 | +1 |
| 50-99 | +2 |
| 20-49 | +3 |
| <20 | +4 |
| Glasgow Coma Scale | |
| 15 | 0 |
| 13-14 | +1 |
| 10-12 | +2 |
| 6-9 | +3 |
| <6 | +4 |
| Bilirubin, mg/dL (μmol/L) | |
| <1.2 (<20) | 0 |
| 1.2-1.9 (20-32) | +1 |
| 2.0-5.9 (33-101) | +2 |
| 6.0-11.9 (102-204) | +3 |
| >12.0 (>204) | +4 |
| Mean arterial pressure or administration (MAP) of vasoactive agents required (mcg/kg/min) | |
| No hypotension | 0 |
| MAP <70mmHg | +1 |
| Dopamine <5 or dobutamine (any dose) | +2 |
| Dopamine >5, epinephrine <0.1, or norepinephrine <0.1 | +3 |
| Dopamine >15, epinephrine >0.1, or norepinephrine >0.1 | +4 |
| Creatinine, mg/dL (μmol/L) (or urine output) | |
| <1.2 (110) | 0 |
| 1.2-1.9 (110-170) | +1 |
| 2.0-3.4 (171-299) | +2 |
| 3.5-4.9 (300-440) or urine output <500mL/day | +3 |
| >5.0 (>440) or urine output <200mL/day | + 4 |
|  |  |

**Supplementary material 1. Questionnaires day 90 and 1 year**

BIOSEP study: 90-day follow up questionnaire

- Readmission
  - “You were discharged from [hospital name] on [date of discharge]. Since then, have you been hospitalized again? (Do not include an overnight stay in the emergency room.)”
    - Yes, No, Unsure
  - [If re-hospitalized]: “About how many nights have you spent in a hospital?
    - Number <1 to maximum logically possible>
  - [If re-hospitalized]: were any of these at a hospital other than [hospitalization name]?
    - Yes, No, Unsure
    - [if Yes]: what was the reason for hospital admission?
- Antibiotics
  - “You were admitted to the emergency department of [hospital name] on [date of presentation]. In the three months leading up to the emergency department visit, did you receive any treatments of antibiotics (either from the general practitioner or specialist?
    - Yes, No, Unsure
  - [If antibiotics prior to presentation]: please specify type, duration, and dosage.
  - “You were discharged from [hospital name] on [date of discharge]. Since then, have you visited the general practitioner?
    - Yes, No, Unsure
  - [If visit to GP]: “About how many times have you visited the general practitioner?
    - Number 1 to maximum logically possible>
  - [If visit to GP]: Did your general practitioner start a new antibiotic treatment? (Do not include antibiotic treatments that were already started during the hospital visit).
    - Yes, No, Unsure
- Symptoms
  - Did you experience any of the following problems IN THE PAST 90 DAYS (1=not at all; 2=a little bit; 3=somewhat; 4=quite a bit; and 5=very much)?
    - Breathing problems
    - Chest pain, chest tightness, or angina
    - A rapid or irregulair heartbeat
    - Swelling in your feet, ankles, or legs when you wake up in the morning
    - Being forced to sleep sitting up in a chair or with at least 3 pillows to prop you up because of shortness of breath
    - Impaired muscle strength
    - Tiredness
    - Memory loss
    - Depressed mood for most of the day
  - Did you experience any of the following problems BEFORE your hospitalization (1=not at all; 2=a little bit; 3=somewhat; 4=quite a bit; and 5=very much)?
    - Breathing problems
    - Chest pain, chest tightness, or angina
    - A rapid or irregulair heartbeat
    - Swelling in your feet, ankles, or legs when you wake up in the morning
    - Being forced to sleep sitting up in a chair or with at least 3 pillows to prop you up because of shortness of breath
    - Impaired muscle strength
    - Tiredness
    - Memory loss
    - Depressed mood for most of the day
- Return to work
  - “Were you employed full-time, part-time, or not at all prior to your hospitalization?”
    - Full-time, part-time, not at all
  - [If previously employed] “Have you been able to return to working?”
    - Yes, No

o [If not returned to work] “Was the reason you have been unable to return to work due to health issues”

- - - Yes, No
  - [If returned to work] “When did you return to work?”
    - Date
  - [If returned to work] “Did you have modified duties upon return to work?”
    - No
    - Yes, check all that apply
      - Reduced work hours
      - Different (modified) work duties
      - Different job altogether
- Return to Normal Activities
  - Have you been about to return to your normal activities?
    - Yes, No
  - On a scale from 1 to 5 (1=without any difficulty; 2= with a little difficulty; 3=with some difficulty; 4=with much difficulty; 5=unable to do so), how much difficulty do you experience with the following daily activities:
    - Chores such as vacuuming or garden work
    - Going up and down stairs at normal pace
    - Walking for at least 15 minutes
    - Running errands and shopping
    - Spelling adequately when writing or typing
    - Reading for at least 15 minutes
- Emotional Impact
  - How much have you been emotionally affected by your health conditions? (options: None

/ Mild / Moderate / Severe / Extreme)

BIOSEP study: 1-year follow-up questionnaire

- Readmission
  - “We had contact with you on [date of 90-day follow-up]. Since then, have you been hospitalized again? (Do not include an overnight stay in the emergency room.)”
    - Yes, No, Unsure
  - [If re-hospitalized]: “About how many nights have you spent in a hospital?
    - Number <1 to maximum logically possible>
  - [If re-hospitalized]: were any of these at a hospital other than [hospitalization name]?
    - Yes, No, Unsure
    - [if Yes]: what was the reason for hospital admission?

**Supplementary material 2. Original protocol**

The BIOSEP study

Towards novel BIOmarkers to diagnose SEPsis on the emergency room

## Amsterdam UMC, The Netherlands:

*Center of Experimental and Molecular Medicine*

# PROTOCOL TITLE

‘Towards novel BIOmarkers to diagnose SEPsis on the emergency room’

| **Protocol ID** | **NL81139.018.22** |
| --- | --- |
| **Short title** | **BIOSEP** |
| **EudraCT number** | NA |
| **Version** | 6 |
| **Date** | 3-4-2024 |
| **Subsidising party** | European Commission (Horizon 2020, Eurostars SEP-RAID) |
| **Pharmacy** | Not applicable |

**TABLE OF CONTENTS**

1. [INTRODUCTION AND RATIONALE 10](#_bookmark0)
2. [OBJECTIVES 12](#_bookmark1)
3. [STUDY DESIGN 13](#_bookmark2)
4. [STUDY POPULATION 14](#_bookmark3)
   1. [Population (base) 14](#_bookmark4)
   2. [Inclusion criteria 14](#_bookmark5)
   3. [Exclusion criteria 15](#_bookmark6)
   4. [Sample size calculation 15](#_bookmark7)
5. [TREATMENT OF SUBJECTS 17](#_bookmark8)
6. [INVESTIGATIONAL PRODUCT 17](#_bookmark9)
7. [NON-INVESTIGATIONAL PRODUCT 17](#_bookmark10)
8. [METHODS 18](#_bookmark11)
   1. [Study parameters/endpoints 18](#_bookmark12)
      1. [Main study parameter/endpoint 18](#_bookmark13)
      2. [Secondary study parameters/endpoints (if applicable) 18](#_bookmark14)
      3. [Other study parameters (if applicable) 18](#_bookmark15)
   2. [Randomisation, blinding and treatment allocation 19](#_bookmark16)
   3. [Study procedures 19](#_bookmark17)
   4. [Withdrawal of individual subjects 22](#_bookmark18)
      1. [Specific criteria for withdrawal (if applicable) 22](#_bookmark19)
   5. [Replacement of individual subjects after withdrawal 22](#_bookmark20)
   6. [Follow-up of subjects withdrawn from treatment 23](#_bookmark21)
   7. [Premature termination of the study 23](#_bookmark22)
9. [SAFETY REPORTING 24](#_bookmark23)
   1. [Temporary halt for reasons of subject safety 24](#_bookmark24)
   2. [AEs, SAEs and SUSARs 24](#_bookmark25)
      1. [Adverse events (AEs) 24](#_bookmark26)
      2. [Serious adverse events (SAEs) 24](#_bookmark27)
      3. [Suspected unexpected serious adverse reactions (SUSARs) 25](#_bookmark28)
   3. [Annual safety report 25](#_bookmark29)
   4. [Follow-up of adverse events 25](#_bookmark30)
   5. [Data Safety Monitoring Board (DSMB) or Safety Committee 25](#_bookmark31)
10. [STATISTICAL ANALYSIS 26](#_bookmark32)
    1. [General considerations 26](#_bookmark33)
    2. [Study parameters objectives 26](#_bookmark34)
    3. [Interim analysis (if applicable) 27](#_bookmark35)
11. [ETHICAL CONSIDERATIONS 28](#_bookmark36)
    1. [Regulation statement 28](#_bookmark37)
    2. [Recruitment and consent 28](#_bookmark38)
    3. [Objection by minors or incapacitated subjects (if applicable) 28](#_bookmark39)
    4. [Benefits and risks assessment, group relatedness 28](#_bookmark40)
    5. [Compensation for injury 29](#_bookmark41)

| [f.](#_bookmark42) | [Incentives (if applicable)](#_bookmark42) | [29](#_bookmark42) |
| --- | --- | --- |
| [12.](#_bookmark43) | [ADMINISTRATIVE ASPECTS, MONITORING AND PUBLICATION](#_bookmark43) | [30](#_bookmark43) |
| [a.](#_bookmark44) | [Handling and storage of data and documents](#_bookmark44) | [30](#_bookmark44) |
| [c.](#_bookmark45) | [Monitoring and Quality Assurance](#_bookmark45) | [30](#_bookmark45) |
| [d.](#_bookmark46) | [Amendments](#_bookmark46) | [31](#_bookmark46) |
| [e.](#_bookmark47) | [Annual progress report](#_bookmark47) | [31](#_bookmark47) |
| [f.](#_bookmark48) | [Temporary halt and (prematurely) end of study report](#_bookmark48) | [31](#_bookmark48) |
| [g.](#_bookmark49) | [Public disclosure and publication policy](#_bookmark49) | [31](#_bookmark49) |
| [13.](#_bookmark50) | [STRUCTURED RISK ANALYSIS](#_bookmark50) | [32](#_bookmark50) |
| [14.](#_bookmark51) | [REFERENCES](#_bookmark51) | [33](#_bookmark51) |

**LIST OF ABBREVIATIONS AND RELEVANT DEFINITIONS**

| **ABR** | **General Assessment and Registration form (ABR form), the application form that is required for submission to the accredited Ethics Committee; in Dutch:**  **Algemeen Beoordelings- en Registratieformulier (ABR-formulier)** |
| --- | --- |
| **AE** | **Adverse Event** |
| **AKI** | **Acute Kidney Injury** |
| **CEMM** | **Center of Experimental Molecular Medicine** |
| **CVVH** | **Continuous Veno-Venous Hemofiltration** |
| **DSMB** | **Data Safety Monitoring Board** |
| **eCRF** | **Electronic Case Report Form** |
| **ED** | **Emergency Department** |
| **EU** | **European Union** |
| **EudraCT** | **European drug regulatory affairs Clinical Trials** |
| **EWS** | **Early Warning Score** |
| **GCP** | **Good Clinical Practice** |
| **GCS** | **Glasgow Coma Scale** |
| **GDPR** | **General Data Protection Regulation; in Dutch: Algemene Verordening**  **Gegevensbescherming (AVG)** |
| **HPLC** | **High-Performance Liquid Chromatography** |
| **HSQC** | **Heteronuclear Single Quantum Coherence** |
| **IC** | **Informed Consent** |
| **ICU** | **Intensive Care Unit** |
| **IL** | **Interleukin** |
| **METC** | **Medical research ethics committee (MREC); in Dutch: medisch-ethische**  **toetsingscommissie (METC)** |
| **MEWS** | **Modified Early Warning Score** |
| **NEWS** | **National Early Warning Score** |
| **NFU** | **Nederlandse Federatie van Universitair Medische Centra** |
| **PCA** | **Principal Component Analysis** |
| **PLS-DA** | **Partial Least Square Discriminant Analysis** |
| **qSOFA** | **Quick Sequential Organ Failure Assessment** |
| **(S)AE** | **(Serious) Adverse Event** |
| **Sponsor** | **The sponsor is the party that commissions the organisation or performance of the research, for example a pharmaceutical**  **company, academic hospital, scientific organisation or investigator. A party** |

|  | **that provides funding for a study but does not commission it is not regarded**  **as the sponsor, but referred to as a subsidising party.** |
| --- | --- |
| **SIRS** | **Systemic Inflammatory Response Syndrome** |
| **SOFA** | **Sequential Organ Failure Assessment** |
| **SUSAR** | **Suspected Unexpected Serious Adverse Reaction** |
| **UAVG** | **Dutch Act on Implementation of the General Data Protection Regulation; in**  **Dutch: Uitvoeringswet AVG** |
| **UMC** | **University Medical Center** |
| **US** | **United States** |
| **WMO** | **Medical Research Involving Human Subjects Act; in Dutch: Wet Medisch-**  **wetenschappelijk Onderzoek met Mensen** |

# SUMMARY

**Rationale:** The international Surviving Sepsis Campaign guidelines have highlighted the need to increase our capacity to timely and accurately diagnose sepsis as the fundamental challenge to the field of infectious diseases. Early diagnosis and treatment of sepsis are critical and associated with decreased mortality rates. However, in everyday clinical practice the administration of for instance antibiotics to a patient with a suspicion of severe infection often takes more than 4 hours after admission. There is an urgent need to rapidly and effectively identify patients with a potential infection on the emergency department (ED) who are at risk of progressing along the infection-sepsis spectrum.

**Objective:** The objectives of the present study are 1) to compare the immune response of patients with or without sepsis presenting to the ED with a(n) (suspected) infection, 2) to determine immune response aberrations that are associated with an increased risk to develop sepsis in patients presenting to the ED with a(n) (suspected) infection without sepsis and 3) to determine the long term cognitive and physical sequelae of sepsis after admission.

**Study design:** Prospective observational cohort study conducted at both locations of the Amsterdam UMC, the Flevoziekenhuis and Sint-Antonius hospital. **Study population:** Adults presenting at the EDs of these hospitals with a(n) (suspected) infection and a MEWS of ≥ 2.

**Intervention (if applicable)**: Not applicable.

**Main study parameters/endpoints:** The main parameters of this study will be SOFA and MEWS score collected on and during admission, diagnosis of sepsis on (ED and/or hospital and/or ICU) admission, final diagnosis and assessment of the host immune response by Raman spectroscopy, transcriptomics, proteomics, metabolomics and gut microbiota. These data will be associated with several clinical parameters, including, but not limited to: duration of hospital stay, duration of ICU stay, hospital mortality, ICU mortality, 28-day mortality, 90-day mortality, 1-year mortality, readmissions and sequelae of sepsis up to 90-days post admission. **Nature and extent of the burden and risks associated with participation, benefit and group relatedness:** The burden and risk associated with participation are negligible. We will in total take 125 ml of blood divided over two time-points, a rectal swab at one time-point and contact participants two times for questionnaires (90-days and 1-year). In a subgroup of participants with an acute respiratory infection, a stool sample will be collected. Participating in this study will not benefit the participants. The knowledge obtained in this study can potentially benefit patients with a(n) (suspected) infection in the future by better predicting which patients are at-risk for poor outcome allowing for the more rapid assessment, diagnosis, and treatment of sepsis.

# INTRODUCTION AND RATIONALE

Despite a global decrease in sepsis burden, sepsis still causes almost 20% of all deaths worldwide [1, 2] and contributes significantly to in-hospital mortality [3]. The surviving sepsis campaign pointed out diagnosis as a fundamental challenge [4]. Early diagnosis and treatment are pivotal and associated with decreased mortality rates [5]. However, in everyday clinical practice taking action upon suspicion of severe infection, e.g. by administering antibiotics, often takes more than 4 hours after admission [6]. In early stages of the septic response, the source of the infection may be unclear and the clinical signs indistinguishable from non- infectious diseases, leading to missed or delayed diagnoses [7].

For over 25 years, patients with an infection who met two or more of the systemic inflammatory response syndrome (SIRS) criteria, were diagnosed with sepsis [8]. The limited specificity of the SIRS criteria led to a redefinition of sepsis in 2016 in which the presence of organ failure, as measured by the sequential organ failure assessment (SOFA) score, in a patient with a (suspected) infection became key [5]. Furthermore, since the SOFA score requires laboratory testing, the quick SOFA (qSOFA) score which is calculated according to three parameters (systolic blood pressure, respiratory rate and Glascow Coma Scale), was introduced to enable early sepsis recognition at bedside. However, the qSOFA unfortunately lacks sensitivity [9]. Early Warning Scores (EWS) also have a poor prognostic value in predicting sepsis mortality [10]. The most recent Surviving Sepsis Campaign guidelines recommend use of SIRS, National Early Warning Score (NEWS) or Modified Early Warning Score (MEWS) as a screening tool for sepsis [4]. The most commonly used are the MEWS and NEWS [11]. To overcome the limitations of clinical scores, several biomarkers for the diagnosis of sepsis have been studied, but so far, none of them have sufficient specificity or sensitivity to be routinely employed in clinical practice [12, 13]. Furthermore, microbiological cultures are time-consuming and only a small percentage of culture tests will yield a positive result for existence of microbes, thus an ideal diagnostic tool for sepsis is not available at this moment [14].

Data from the United States (US) indicate that sepsis is a common presentation in the emergency department (ED) and represents roughly one third of all hospital admissions that culminate in death [15]. In addition, one Finnish study from 2011 has estimated that up to 50% of ED presentations for a suspected infection (defined by the physician’s decision to take

samples for blood cultures) have sepsis with a 28-days mortality rate of up to 30% [16]. Patients with hospital-acquired sepsis are even three times more at risk for in-hospital mortality compared to patients with community-acquired sepsis [14].

After hospital discharge patients who were initially admitted for sepsis still have an increased risk of death [17]. Data from the US showed that one-third of sepsis survivors die during the following year, of which half of the deaths were related to complications of sepsis.

Furthermore, one-sixth of sepsis survivors did experience severe persistent physical disability or cognitive impairment [18]. As a result, the Surviving Sepsis Campaign guidelines now recommend assessment and follow-up for physical, cognitive, and emotional problems of patients after hospital discharge for sepsis [4]. Although the challenges of critical illness survivorship are now increasingly well documented, there are relatively few studies on enhancing recovery and how to identify patients at increased risk [17].

There is a high need to rapidly and effectively identify patients on the ED who are at risk of progressing along the infection-sepsis spectrum. The objectives of the present study are 1) to compare the immune response of patients with or without sepsis presenting to the ED with (suspected) infection, 2) to determine immune response aberrations that are associated with an increased risk to develop sepsis in patients presenting to the ED with (suspected) infection without sepsis and 3) to determine the long term cognitive and physical sequelae of sepsis after admission. We will investigate the immune response using Raman spectroscopy [19, 20] and genomic, transcriptomic, metabolomic and proteomic data in order to better stratify sepsis patients and gain insight into the pathophysiology of sepsis. The hypothesis is that Raman spectroscopy can be used as a classifier of immunological profiles that could allow to identify a fraction of the population with a high likelihood of developing sepsis. Raman spectroscopy is a non-destructive analytical technique that uses the inelastic scattering of light to provide information on chemical composition. Raman spectroscopy in unbiased in terms of detection and allows to capture the combined response of samples. Raman spectroscopy can determine changes or fluctuations in elements of the immune system, alerting about a possible infection.

# OBJECTIVES

Objectives:

1. To compare the immune response of patients with or without sepsis presenting to the ED with a(n) (suspected) infection.
2. To determine immune response aberrations that are associated with an increased risk of developing sepsis in patients presenting to the ED with a(n) (suspected) infection without sepsis.
3. To determine the long term cognitive and physical sequelae of sepsis after admission.

**Sepsis will be defined in accordance with the current Sepsis 3.0 criteria** as a(n) (suspected) infection with evidence of organ failure, as reflected by a SOFA (Sequential Organ Failure Assessment) score of ≥2 [5]. Notably, a molecular definition of sepsis does not exist and there is no pathological gold standard; therefore, in accordance with the current international consensus [5], we consider the commonly used clinical organ failure (SOFA) criteria the best option. The SOFA score is composed of six organ dysfunctions (cardiovascular, pulmonary, renal, hepatic, coagulation and neurological) [21]. The SOFA score was developed for ICU patients, but its components can be easily scored in an ED (and hospital ward) setting with the exception of the pulmonary component; this pulmonary dysfunction score is based on the PaO2/FiO2 (PF) ratio, wherein PaO2 is the partial pressure of oxygen in arterial blood and FiO2 the fraction of inspired oxygen. Measurement of the PaO2 requires an arterial blood puncture, which is not routinely done on the ER or hospital ward.

Therefore, we will use an alternative method to determine the respiratory SOFA by determining the SpO2/FiO2 (SF) ratio, wherein SpO2 is peripheral oxygen saturation [22]. SpO2 is routinely measured by finger pulse oximeter in patients with suspected infection; FiO2 is 21% when breathing room temperature and increases by 4% with each liter of oxygen provided per minute to a patient via a nasal cannula. Cut-off values for SF ratios correlating with SOFA pulmonary scores based on PF ratios have been validated in large data sets [22].

# STUDY DESIGN

Prospective observational cohort study conducted at both locations of the Amsterdam UMC, the Flevoziekenhuis and Sint-Antonius hospital. We expect to include a maximum of 3330 patients in 48 months.

Adults presenting at the ED of one of aforementioned hospitals with a(n) (suspected) infection and a Modified Early Warning Scores (MEWS) of ≥ 2 will be screened for eligibility for this study.

# STUDY POPULATION

## Population (base)

The research population will include adults aged ≥ 18 years, who are admitted to the ED with a (suspected) infection and a MEWS of ≥ 2.

## Inclusion criteria

In order to be eligible to participate in this study, a subject must meet all of the following criteria:

- Age ≥ 18 years;
- Presentation at the ED;
- Clinical suspicion of infection;
- MEWS of ≥ 2

| **MEWS Score** | **3** | **2** | **1** | **0** | **1** | **2** | **3** |
| --- | --- | --- | --- | --- | --- | --- | --- |
| **Respiratory rate (breaths/min)** |  | <9 |  | 9-14 | 15-20 | 21-30 | >30 |
| **Saturation with therapy (%)** | <90 |  |  |  |  |  |  |
| **Heartrate (beats/min)** |  | <40 | 40-50 | 51-100 | 101-110 | 111-130 | >130 |
| **Systolic blood pressure (mmHg)** | <70 | 70-80 | 81-100 | 101-200 |  | >200 |  |
| **Urine production (mL)** |  |  | <75ml in the last 4 hours |  |  |  |  |
| **Consciousness** |  |  |  | Alert | Response to addressing | Response to pain | Unresponsive |
| **Temperature (degrees Celsius)** |  | <35.1 | 35-1-36-5 | 36.6-37.5 | >37.5 |  |  |
| **Worries** |  |  | If you are worried about the patient’s condition |  |  |  |  |

## Exclusion criteria

A potential subject who meets the following criteria will be excluded from participation in this study:

- No informed consent is provided by patient or its legal representative.

## Sample size calculation

Since we aim to provide insight into the immune response of patients with a (suspected) infection with or without sepsis, we carried out power analysis for three biomarkers (interleukin (IL)-9, IL12 and IL-17) using mean biomarker levels in admission laboratory values of patients with SIRS versus sepsis [23]. With a power of 80% to detect difference, employing a two-sided Z-test for two means with an alpha of 0.05 and assuming a sample size ratio of 0.1 between the two groups, a sample size of 91 and 328 depending on the biomarker is required in the group with sepsis and of 906 and 3270 in the group without sepsis. The sample size ratio of

0.1 will be explained below.

The Amsterdam UMC team conducted a survey on the ED of both locations (Academic Medical Center and Free University Hospital) encompassing a 60-day period on week days (Monday to Friday) between November 2019 and February 2020 (i.e., prior to the COVID-19 pandemic). In this period 7538 ED presentations occurred, of which 279 with suspected infection and a MEWS score ≥3 (patients with lower MEWS scores were not included in this pilot study). Thirty patients (11%) were diagnosed with sepsis (based on a SOFA score ≥ 2). Extrapolation of these data to expected patient numbers in a one-year period results in 1674 ER presentations for infection with a MEWS score ≥ 3 to both centers, of which 180 with a diagnosis of sepsis.

The survey also showed that the overall population presented to the ED with a MEWS score

≥2 is twice as large as the overall population with a MEWS score ≥3. Assuming that the proportion of suspected infections remains approximately the same, it can be expected that if an inclusion criterion of suspected infection with a MEWS score ≥2 is used, the number of patients presented at the ED will be >3300 annually (including approximately 300 patients with sepsis), with a higher likelihood of capturing “pre-sepsis” patients.

Based on the survey summarized above patients will prospectively be included that present to the ER with a suspected infection and a MEWS score ≥2.

Given the size of our research team (on the ED and in the laboratory) we will be able to include a maximum of approximately 15 patients per week. With this strategy we expect to enroll 330 patients with sepsis in four years.

# TREATMENT OF SUBJECTS

Not applicable.

# INVESTIGATIONAL PRODUCT

Not applicable.

# NON-INVESTIGATIONAL PRODUCT

Not applicable.

# METHODS

## Study parameters/endpoints

## Main study parameter/endpoint

- SOFA and MEWS score collected on and during admission;
- Sepsis at (ED and/or hospital and/or ICU) admission;
- Final diagnosis for hospitalization;
- Assessment of the host immune response by Raman spectroscopy, transcriptomics, proteomics, metabolomics, lipidomics, and gut microbiota.

## Secondary study parameters/endpoints (if applicable)

- Duration of hospital stay;
- Duration of ICU stay;
- Hospital mortality;
- ICU mortality;
- 28-day mortality;
- 90-day mortality;
- 1-year mortality;
- Sequelae of sepsis up to 90-days post admission (decreased executive functions, weakness, fatigue, mood, etc.);
- All-cause readmissions in the first year after discharge.

## Other study parameters (if applicable)

- General information: sex, age, height, weight, race, ethnicity, medical history, medication use prior to admission, antibiotic exposure in the 3 months prior to admission, post-surgical patient;
- Information collected on and during admission: laboratory values, organ support, vital signs, sedation, Glascow Coma Scale (GCS);
- Duration of symptoms prior to ED presentation;
- Site of infection;
- Source control assessment after diagnosis;
- Microbiology tests (culture, PCR, pneumococcal antigen, etc.) for causative microorganisms;
- Therapy since admission: antibiotic treatment, fluid administration, inotropic/vasopressin drugs, indication for therapeutic anticoagulation, type of therapeutic anticoagulation;
- Ceiling of care decisions;
- Outcomes: venous thromboembolic events, arterial thrombotic events (including acute myocardial infarction), bleeding complications, disseminated intravascular coagulation (DIS), acute kidney injury (AKI), need for continuous veno-venous hemofiltration (CVVH) / dialysis / mechanical ventilation / extracorporeal membrane oxygenation (ECMO), discharge location.

## Randomisation, blinding and treatment allocation

Not applicable.

## Study procedures

Screening and eligibility assessment

The caretaking team on the ED of potential study participants will be informed about the possibility of their patient to partake in this study. Potential participants will then be identified by a member of their immediate care team and asked for verbal consent to be approached by research staff who are not members of their care team. If patients are incapacitated during the screening process, consent will be asked from the patients’ legal representative. If the patient or its legal representative agrees, the verbal consent will be recorded in the participants’ medical record. A ‘Screening Log’ will be maintained of all the patients who undergo screening regardless of whether they decide to participate in the study.

Recruitment and informed consent

Patients will be recruited by one of the executive investigators or research nurses (GCP- trained study staff) by screening the patients for the presence of an infection and a MEWS

≥ 2. Treating physicians will be informed about the study. If possible, informed consent will be asked by the investigator and signed by patient and investigator. Written and verbal versions of the Participant Information Sheet and Informed Consent will detail the exact nature of the study; the implications and constraints of the protocol; and any risks involved in taking part. It will be clearly stated that the participant is free to withdraw from the study at any time for any reason without prejudice to future care, and with no obligation to give a reason for withdrawal. Participant Information Sheets will be available in Dutch and English. The person obtaining consent must be suitably qualified and experienced, and be authorized to do so by the Principal Investigator. A copy of the signed Informed Consent will be given to the participants. The original signed form will be retained at the study site. If blood is drawn for standard care when the patient arrives in the ED, and it is not feasible for

research staff to speak to the patient before the draw, a member of the care team will ask

the patient for verbal consent to draw extra blood for the study samples. Regular information and training sessions will be held by research staff to ensure adequate knowledge and training of ED care providers, so that they will be able to inform patient adequately about the study and its implications. Subsequently, a member of the research staff will always formally recruit and inform the patient and obtain written consent, per the operating procedure mentioned above. Obtaining verbal consent through the care team and drawing all the blood in one time will prevent multiple blood draws being needed and thus minimize burden for the patient.

Incapacitated patients

When the patient is unable or incapable of giving informed consent (e.g. due to loss of consciousness) informed consent will be asked from the legal representative of the patients, preferably in person, but if necessary by telephone. If consent is given by telephone, the researchers will make an appointment with the legal representative to sign the informed consent form as soon as possible. The researchers will contact the patient or next-of-kin to provide information about the study, its aim and the burden to the patient.

When the patient regains consciousness, he/she will also be asked informed consent. A significant proportion of patients with sepsis have reduced consciousness as a manifestation of the disease. These are usually patients with a severe course [24].

Excluding this group will bias the results. This group of patients may actually benefit more from better recognition of the syndrome.

Baseline assessments and subsequent visits

Following provision of consent, baseline data will be collected from patient medical charts. Blood samples, rectal swab and information on treatment and care will be collected. We will draw blood twice in our included patients, 67,5 ml of blood once on the ED and once 57,5 ml 4 (3-5) days later if patients are still admitted to the hospital. We will also collect a rectal swab on the ED. We aim to perform the blood sampling together with blood sampling needed for standard care. In the days after admission, vital signs and clinical outcomes will be recorded to determine our previous stated objectives. Patients will be followed up till the day of discharge or mortality.

Venous blood samples

Blood samples will be collected by a trained phlebotomist with the subject either seated in an upright position or lying down in bed. Subsequently, blood samples will be centrifuged

and stored at -80°C in the Amsterdam UMC biobank. At each visit no more than 67,5 ml of venous blood will be taken. An overview of the schedule is shown below in Table 1.

## Table 1. Overview of blood samples

|  | **Day 0** | **Day 4 (3-5)** |
| --- | --- | --- |
| Plasma and serum | 65 ml | 55 ml |
| PAXgene tube | 2,5 ml | 2,5 ml |
|  |  |  |
| **Total** | **67,5 ml** | **57,5 ml** |

Given the size of our research team (on the ED and in the laboratory) and our main interest in the visits on the ED, the scheduled visit on day 4 will take place if this is logistically feasible.

Rectal swabs

Rectal swabs (FLOQSwabs 552C, Copan, CA, USA) will be collected on day 0 and used for microbiota analysis. This validated sample method is more reproducible and reliable compared to the collection of feces samples. (32) The swabs are inserted into the anal canal by the research team member, treating nurse or the participant itself, beyond the anal verge (±3 cm). Rectal swabs will be deposited in a container with 500 µl. Reduced Transport Fluid (RTF) buffer (33) and may be kept at room temperature for 2 hours prior to storage at −80°C.

Laboratory procedures

The immune response will be evaluated using various readouts:

- Raman spectroscopy [19, 20], a non-destructive analytical technique that uses the inelastic scattering of light to provide information on chemical composition, is unbiased in terms of detection and allows to capture the combined response of samples. Raman spectroscopy can determine changes or fluctuations in elements of the immune system, alerting about a possible infection. The hypothesis is that Raman spectroscopy can be used as a classifier of immunological profiles that could allow to identify a fraction of the population with a high likelihood of developing sepsis. We will investigate plasma samples applying Raman spectroscopy and different measurement parameters. For the Raman analysis blood preserved at -80°C is needed.
- Genetics data. A first set of data will be provided by analysis of the genetic variants influencing the clinical outcome. A genome-wide SNP array platform (HumanOmniExpressExome-8 array) will identify common polymorphisms that
- influence clinical and immunological outcome.
- Transcriptome platform. An important component of the data to be collected is represented by the assessment of gene transcription profiles. RNA-sequencing technology will be used in a targeted subgroup of patients with adverse outcome. . In addition, blood leukocytes will be viably frozen for later analysis using single-cell RNA- sequencing in a subset of patients, in order to identify subpopulations of immune cells that may be responsible for the adverse outcome in sepsis.
- Metabolome and proteomics platforms. MS-based metabolomics platform to assess the broad metabolic profiles in plasma of patients will be based on high-performance

liquid chromatography (HPLC) and by 1H-NMR and 2D 13C-1H- heteronuclear single quantum coherence (HSQC) spectroscopy in a UltrashiedTM 800 Plus (Bruker). This methodology will permit the assessment of both hydrophile and hydrophobe metabolites. In addition, a focused proteomics assessment of inflammatory mediators will be initiated using Olink technology. Moreover, next to bulk proteomic analyses, single cell proteomics analyses will be done on selected patients using mass cytometry on viably frozen blood leukocytes; this part will focus on Cell signaling implicated in innate immunity (e.g., pNFĸB, Iĸ-Bα, pP38, pERK1/2, pAkt, pAMPK, p4EBP1 and pS6), as described [25, 26].

- Gut microbiota. Rectal swabs are stored in 70% ethanol contained in a weighed glass centrifuge tube with a screw cap prior to storage at -80°C. Microbiota sequencing will be performed as earlier described by our group [27]. In brief, DNA will be extracted using a repeated bead beating protocol and bacterial microbiota are characterized by a 16S rRNA gene sequencing targeting the V3- V4 region.

Questionnaires

On day 90 and at one year, short validated questionnaires regarding mortality, readmissions, healthcare use and the sequelae of sepsis will be taken. The questionnaires will be obtained within a timeframe of two weeks surrounding day 90 and one month surrounding the one year mark. The questionnaires consist of questions of two validated questionnaires combined [28, 29]. The questionnaires allow us to assess the effects of sepsis and a hospital admission for this disease in a systemic manner. We have added a copy of the questionnaires in the attachments (S1).

## Withdrawal of individual subjects

Patients or its legal representatives are free to withdraw consent at any time without providing a reason. Patients or legal representatives who wish to withdraw consent for the study will have anonymized data and samples collected up to the point of that withdrawal of consent included in the analyses. The patient will not contribute further data to the study.

Data up to the time of withdrawal will be included in the analyses unless the patient explicitly states that this is not their wish.

## Specific criteria for withdrawal (if applicable)

Not applicable.

## Replacement of individual subjects after withdrawal

If a patients withdraws informed consent, he or she will be replaced by a new subject.

## Follow-up of subjects withdrawn from treatment

Not applicable.

## Premature termination of the study

Not applicable, given the observational nature of the study.

# SAFETY REPORTING

## Temporary halt for reasons of subject safety

In accordance to section 10, subsection 4, of the WMO, the sponsor will suspend the study if there is sufficient ground that continuation of the study will jeopardize subject health or safety. The sponsor will notify the accredited METC without undue delay of a temporary halt including the reason for such an action. The study will be suspended pending a further positive decision by the accredited METC. The investigator will take care that all subjects are kept informed.

## AEs, SAEs and SUSARs

## Adverse events (AEs)

Adverse events are defined as any undesirable experience occurring to a subject during the study, whether or not considered related to trial procedure. All adverse events reported spontaneously by the subject or observed by the investigator or his staff will be recorded.

## Serious adverse events (SAEs)

A serious adverse event is any untoward medical occurrence or effect that

- results in death;
- is life threatening (at the time of the event);
- requires hospitalisation or prolongation of existing inpatients’ hospitalisation;
- results in persistent or significant disability or incapacity;
- is a congenital anomaly or birth defect; or
- any other important medical event that did not result in any of the outcomes listed above due to medical or surgical intervention but could have been based upon appropriate judgement by the investigator.

An elective hospital admission will not be considered as a serious adverse event.

As we propose an observational study, we will only report AEs and SAEs that are related to blood sampling. Standard clinical care will not be affected or hampered by participation in the study. We do not expect AEs and SAEs as the result of blood sampling.

The investigator will report all SAEs to the sponsor without undue delay after obtaining knowledge of the events.

The sponsor will report the SAEs through the web portal *ToetsingOnline* to the accredited METC that approved the protocol, within 7 days of first knowledge for SAEs that result in death or are life threatening followed by a period of maximum of 8 days to complete the initial preliminary report. All other SAEs will be reported within a period of maximum 15 days after the sponsor has first knowledge of the serious adverse events.

## Suspected unexpected serious adverse reactions (SUSARs)

Not applicable.

## Annual safety report

Not applicable.

## Follow-up of adverse events

All AEs will be followed until they have abated, or until a stable situation has been reached. Depending on the event, follow up may require additional tests or medical procedures as indicated, and/or referral to the general physician or a medical specialist.

SAEs need to be reported till end of study within the Netherlands, as defined in the protocol.

## Data Safety Monitoring Board (DSMB) or Safety Committee

Not applicable due to negligible risks of our study.

# STATISTICAL ANALYSIS

## General considerations

Demographic and clinical data will be expressed as mean with the standard deviation for variables with a parametric distribution and median plus interquartile range for variables with a non-normal distribution. Variable distribution will be assessed visually by histograms and QQ-plots. Depending on data distribution groups of sepsis patients will be compared using either parametric or non-parametric statistical tests. Multivariate models will be evaluated by linear regression. Missing data on risk factors will be handled by multiple imputation analyses when data is missing at random or completely at random. Type-1 error will be controlled with Bonferroni correction multiple testing correction. Throughout a test probability (p) < 0.05 will be considered significant.

These analyses will be performed within the R environment (R Development Core Team (2008). R: A language and environment for statistical computing. R Foundation for Statistical Computing, Vienna, Austria).

## Study parameters objectives

The data will be analyzed according to sepsis or non-sepsis groups and/or some other classifications based on the pathophysiology. To account for dependencies in repeatedly measured observations within a subject, a linear mixed-effect model will be used to assess the differences between the groups. Multivariate data analysis including unsupervised principal component analysis (PCA), and supervised partial least square discriminant analysis (PLS-DA) will be performed. PCA will be used to obtain a general overview of the immunologic profile and to detect outliers. PLS-DA will be performed to separate different groups. The quality of the model will be described by the parameter of R2 and Q2 which represent model fitness and predictive ability. All PLS-DA models will be validated using the permutation test.

Using the Raman spectra captured from the samples, a classification system will be developed for the stratification of samples and its performance will be assessed. Samples will be divided in two subgroups. The first will be used for data analysis and system training, allowing to extract patterns that can be used for patient stratification. The second subgroup will be used to test, whether the predictions provided by the system are correct and consistent. The data will be analyzed using multivariate analysis; and both supervised

(support vector machine (SVM), neural networks) and non-supervised (Principal Component Analysis, Hierarchical Component Analysis) techniques will be employed.

Several markers for coagulation, inflammation and endothelial activation will be measured. Quantification will be done with a custom Luminex assay.

Differences in microbiota community composition between patient groups (e.g. patients with and without sepsis, or patients with and without long term sequelae of sepsis) will be assessed using permutational multivariate analysis of variance (β-diversity using the weighted UniFrac distance). To identify community members driving these differences, we will use random forest classifier analysis. Alpha diversity will be assessed by calculating the Shannon Diversity Index. Finally, we aim to link microbiota composition to immune responses through permutation ANOVA, as earlier described [30].

## Interim analysis (if applicable)

Not applicable.

# ETHICAL CONSIDERATIONS

## Regulation statement

The study will be conducted according to the principles of the Declaration of Helsinki (as approved on the 64th WMA General Assembly, Fortaleza, Brazil, October 2013, retrieved via: https://[www.wma.net/policies-post/wma-declaration-of-helsinki-ethical-principles-](http://www.wma.net/policies-post/wma-declaration-of-helsinki-ethical-principles-) for_medical-research-involving-human-subjects/) and in accordance with the Medical Research Involving Human Subjects Act (WMO) and other (institutional) guidelines, regulations and acts.

## Recruitment and consent

Subjects will be informed of the study by the treating physician, study coordinator, informed colleague and/or research nurse. If they are interested, they will be given the patient information letter, and the principle investigator/trial nurse will be summoned to visit the patient to discuss all questions patients might have. Informed consent will be obtained if the patient is eligible for the study and wants to participate in the study. Subject will be given a maximum of 24 hours of consideration.

## Objection by minors or incapacitated subjects (if applicable)

When the patient is unable or incapable of giving informed consent (e.g. due to loss of consciousness), informed consent will be asked from the legal representative of the patients, preferably in person, but if necessary by telephone. The researchers will contact the patient or next-of-kin to provide information about the study, its aim and the burden to the patient. When the patient regains consciousness, he/she will also be asked informed consent.

## Benefits and risks assessment, group relatedness

Patients have no benefit of participation in the study. Patients receive their regular treatment as was intended and this will not be influenced by the study. The burden of the blood samples taken is negligible. There is no additional risk in participating in the study. Patients participating in this study will be subjected twice to a blood withdrawal of a maximum of 67,5 ml, once to a rectal swab and twice to a questionnaire (90-day and 1-year follow-up). Participation will contribute to the improvement of the knowledge about sepsis.

## Compensation for injury

The sponsor/investigator has a liability insurance which is in accordance with article 7 of the WMO.

Because of the observational nature of this study and the extremely small risk of SAEs, the METC is asked to grant dispensation from the statutory obligation to provide insurance to cover damage to research subjects caused by the study in accordance with article 7 of the WMO.

## Incentives (if applicable)

Not applicable.

# ADMINISTRATIVE ASPECTS, MONITORING AND PUBLICATION

## Handling and storage of data and documents

Data will be handled confidentially and coded. To ensure data security and to protect privacy, data on individual subjects will be encoded according to a subject identification code list. The key to the code will be safeguarded by both the executive and coordinating investigators. They will have access to the data at any time. The research file will be password protected and stored for 15 years. The blood samples will be stored for 5 years. The study will be reported to the ‘data protection officer’ of the Amsterdam UMC. The handling of personal data complies with the EU General Data Protection Regulation and the Dutch Act on Implementation of the General Data Protection Regulation (in Dutch: Uitvoeringswet AVG, UAVG). The local investigator site file and central master file and the electronic data from the electronic Case Report Form (eCRF) will be stored for 15 years. All information, data, and results that originate from this study may not be disclosed without the written permission of the principal investigator.

## Future use of samples

Samples collected will be used for the purpose of BIOSEP and stored for a maximum of 5 years for future analyses that are relevant to the study questions. The standard consent form will request consent from patients for sample storage and/or export of samples to a collaborating institution for investigations that cannot be performed locally. This collaborating institution is called DeepUll. A biotech company that focuses its activity on the development of a new diagnostic system for early-stage sepsis condition. This company can perform the measurements using Raman Spectometry [19, 20] (explained in the introduction and laboratory procedures). Any proposed plans to use samples other than for those relevant to the study question will be submitted to the relevant ethics committees prior to any testing.

## Monitoring and Quality Assurance

The study will be monitored by the clinical monitoring center. According to the

applicable Nederlandse Federatie van Universitair Medische Centra (NFU)-guidelines the chance of additional burden and risks for the patient are negligible, so monitoring will be minimal. What this entails, can be found on page 41 and 42 of the Guideline Quality Assurance of Research Involving Human Subjects of the NFU [31].

## Amendments

Amendments are changes made to the research after a favourable opinion by the accredited METC has been given. All amendments will be notified to the METC that gave a favourable opinion.

All substantial amendments will be notified to the METC and to the competent authority.

Non-substantial amendments will not be notified to the accredited METC and the competent authority, but will be recorded and filed by the sponsor.

## Annual progress report

The sponsor/investigator will submit a summary of the progress of the trial to the accredited METC once a year. Information will be provided on the date of inclusion of the first subject, numbers of subjects included and numbers of subjects that have completed the trial, serious adverse events/ serious adverse reactions, other problems, and amendments.

## Temporary halt and (prematurely) end of study report

The investigator/sponsor will notify the accredited METC of the end of the study within a period of 8 weeks. The end of the study is defined as the last patient’s last visit.

The sponsor will notify the METC immediately of a temporary halt of the study, including the reason of such an action.

In case the study is ended prematurely, the sponsor will notify the accredited METC within 15 days, including the reasons for the premature termination.

Within one year after the end of the study, the investigator/sponsor will submit a final study report with the results of the study, including any publications/abstracts of the study, to the accredited METC.

## Public disclosure and publication policy

The results of this study will be disclosed unreservedly and published in a peer reviewed medical journal.

# STRUCTURED RISK ANALYSIS

Not applicable.

# REFERENCES

1. Rudd, K.E., et al., *Global, regional, and national sepsis incidence and mortality, 1990– 2017: analysis for the Global Burden of Disease Study.* The Lancet, 2020. **395**(10219):

p. 200-211.

1. van der Poll, T., M. Shankar-Hari, and W.J. Wiersinga, *The immunology of sepsis.*

Immunity, 2021. **54**(11): p. 2450-2464.

1. Liu, V., et al., *Hospital deaths in patients with sepsis from 2 independent cohorts.*

JAMA, 2014. **312**(1): p. 90-2.

1. Evans, L., et al., *Surviving sepsis campaign: international guidelines for management of sepsis and septic shock 2021.* Intensive Care Med, 2021. **47**(11): p. 1181-1247.
2. Singer, M., et al., *The Third International Consensus Definitions for Sepsis and Septic Shock (Sepsis-3).* JAMA, 2016. **315**(8): p. 801-10.
3. Seymour, C.W., et al., *Assessment of Clinical Criteria for Sepsis: For the Third International Consensus Definitions for Sepsis and Septic Shock (Sepsis-3).* JAMA, 2016. **315**(8): p. 762-74.
4. Klein Klouwenberg, P.M., et al., *Likelihood of infection in patients with presumed sepsis at the time of intensive care unit admission: a cohort study.* Crit Care, 2015. **19**: p. 319.
5. Bone, R.C., et al., *Definitions for sepsis and organ failure and guidelines for the use of innovative therapies in sepsis. The ACCP/SCCM Consensus Conference Committee. American College of Chest Physicians/Society of Critical Care Medicine.* Chest, 1992. **101**(6): p. 1644-55.
6. Liu, Y.C., et al., *Quick Sequential Organ Failure Assessment as a prognostic factor for infected patients outside the intensive care unit: a systematic review and meta- analysis.* Intern Emerg Med, 2019. **14**(4): p. 603-615.
7. Hamilton, F., et al., *Early Warning Scores do not accurately predict mortality in sepsis: A meta-analysis and systematic review of the literature.* J Infect, 2018. **76**(3): p. 241- 248.
8. Azijli, K., Minderhoud, T., Mohammadi, P., Dekker, R., Brown, V., Attaye, T., Huisman,

S. J., Hettinga-Roest, A. A., & Nanayakkara, P, *A prospective, observational study of the performance of MEWS, NEWS, SIRS and qSOFA for early risk stratification for adverse outcomes in patients with suspected infections at the emergency department.* Acute medicine, 2021. **20(2)**: p. 116–124.

1. Pierrakos, C. and J.L. Vincent, *Sepsis biomarkers: a review.* Crit Care, 2010. **14**(1): p. R15.
2. van Engelen, T.S.R., et al., *Biomarkers in Sepsis.* Crit Care Clin, 2018. **34**(1): p. 139- 152.
3. Rothman, M., et al., *Sepsis as 2 problems: Identifying sepsis at admission and predicting onset in the hospital using an electronic medical record-based acuity score.* J Crit Care, 2017. **38**: p. 237-244.
4. Rhee, C., et al., *Incidence and Trends of Sepsis in US Hospitals Using Clinical vs Claims Data, 2009-2014.* JAMA, 2017. **318**(13): p. 1241-1249.
5. Uusitalo-Seppala, R., et al., *Early detection of severe sepsis in the emergency room: diagnostic value of plasma C-reactive protein, procalcitonin, and interleukin-6.* Scand J Infect Dis, 2011. **43**(11-12): p. 883-90.
6. Prescott, H.C., J.B. Sussman, and W.J. Wiersinga, *Postcritical illness vulnerability.*

Curr Opin Crit Care, 2020. **26**(5): p. 500-507.

1. Prescott, H.C. and D.C. Angus, *Enhancing Recovery From Sepsis: A Review.* JAMA, 2018. **319**(1): p. 62-75.
2. Arend, N., et al., *Detection and Differentiation of Bacterial and Fungal Infection of Neutrophils from Peripheral Blood Using Raman Spectroscopy.* Anal Chem, 2020. **92**(15): p. 10560-10568.
3. Neugebauer, U., et al., *Fast differentiation of SIRS and sepsis from blood plasma of ICU patients using Raman spectroscopy.* Journal of Biophotonics, 2014. **7**(3-4): p. 232- 240.
4. Vincent, J.L., Moreno, R., Takala, J., Willatts, S., De Mendonça, A., Bruining, H., Reinhart, C. K., Suter, P. M., & Thijs, L. G. On behalf of the Working Group on Sepsis- Related Problems of the European Society of Intensive Care Medicine., *The SOFA (Sepsis-related Organ Failure Assessment) score to describe organ dysfunction/failure.*

*.* Intensive care medicine, 1996. **22(7)**: p. 707–710.

1. Pandharipande, P.P., et al., *Derivation and validation of Spo2/Fio2 ratio to impute for Pao2/Fio2 ratio in the respiratory component of the Sequential Organ Failure Assessment score*.* Critical Care Medicine, 2009. **37**(4): p. 1317-1321.
2. Jekarl, D.W., et al., *Diagnosis and evaluation of severity of sepsis via the use of biomarkers and profiles of 13 cytokines: a multiplex analysis.* Clin Chem Lab Med, 2015. **53**(4): p. 575-81.
3. Sonneville, R., Verdonk, F., Rauturier, C., Klein, I. F., Wolff, M., Annane, D., Chretien, F., & Sharshar, T, *Understanding brain dysfunction in sepsis.* Annals of intensive care, 2013. **3**(15).
4. Baskar, R., et al., *TRAIL-induced variation of cell signaling states provides nonheritable resistance to apoptosis.* Life Sci Alliance, 2019. **2**(6).
5. Hartmann, F.J., et al., *Single-cell metabolic profiling of human cytotoxic T cells.* Nat Biotechnol, 2021. **39**(2): p. 186-197.
6. Haak, B.W., et al., *Bacterial and Viral Respiratory Tract Microbiota and Host Characteristics in Adults With Lower Respiratory Tract Infections: A Case-Control Study.* Clin Infect Dis, 2022. **74**(5): p. 776-784.
7. Huang, C.Y., et al., *Life after sepsis: an international survey of survivors to understand the post-sepsis syndrome.* Int J Qual Health Care, 2019. **31**(3): p. 191-198.
8. Chopra V, F.S., O'Malley M, Malani AN, Prescott HC, *Sixty-Day Outcomes Among Patients Hospitalized With COVID-19.* Ann Intern Med, 2021 **Apr;174(4)**: p. 576-578.
9. Schirmer, M., et al., *Linking the Human Gut Microbiome to Inflammatory Cytokine Production Capacity.* Cell, 2016. **167**(4): p. 1125-1136 e8.
10. *Guideline Quality assurance of research involving human subjects dec20*. 2020 Accessed on November 3, 2021]; Available from: [https://www.nfu.nl/sites/default/files/2021-](https://www.nfu.nl/sites/default/files/2021-01/21.00024_Guideline_Quality_assurance_of_research_involving_human_subjects_dec20_0.pdf) [01/21.00024_Guideline_Quality_assurance_of_research_involving_human_subjects_d](https://www.nfu.nl/sites/default/files/2021-01/21.00024_Guideline_Quality_assurance_of_research_involving_human_subjects_dec20_0.pdf) [ec20_0.pdf](https://www.nfu.nl/sites/default/files/2021-01/21.00024_Guideline_Quality_assurance_of_research_involving_human_subjects_dec20_0.pdf).
