# Supplementary material for: Towards novel BIOmarkers to diagnose SEPsis (BIOSEP) in the emergency room: a protocol for a multicentre, prospective cohort study
Source: BMJ Open. 2025 Aug 1;15(7):e103138. doi: 10.1136/bmjopen-2025-103138 (PMC12314967; doi:10.1136/bmjopen-2025-103138)
Supplement: Supplementary file 2 [file bmjopen-2025-103138.reviewer_comments.pdf]

PEER REVIEW HISTORY

BMJ Open publishes all reviews undertaken for accepted manuscripts. Reviewers are asked to complete a checklist review form and are provided with free text boxes to elaborate on their assessment. These free text comments are reproduced below.

ARTICLE DETAILS

Title (Provisional)

Towards novel BIOmarkers to diagnose SEPsis on the emergency room (BIOSEP): protocol for a multicentre, prospective cohort study

Authors

Reijnders, Evelien; Turgman, Oren; Joosten, Sebastiaan C.M.; Schinkel, Michiel; Slim, Marleen A; Douma, Renee; Moeniralam, Hazra; Peters-Sengers, Hessel; van der Poll, Tom; Wiersinga, Joost

---

VERSION 1 - REVIEW

**Reviewer**	**1**
**Name**	**Trongtrakul, Konlawij**
**Affiliation**	**Chiang Mai University Faculty of Medicine, Internal Medicine**
**Date**	**24-Jun-2025**
**COI**	**None**

The authors present a well-designed, prospective, multicentre cohort protocol that aims to explore early host immune responses and identify novel biomarkers to improve sepsis diagnosis in the emergency department (ED) setting by the integration of transcriptomics, proteomics, metabolomics, and microbiota profiling. This study is ambitious and aligns well with the goal of advancing precision medicine in sepsis care. The study is methodologically sound and addresses a critical knowledge gap.

I would like to commend the team for the thorough planning and comprehensive methodology. Below are a few suggestions to consider for clarity and completeness:

1. The current version is well written and may not require revision. However, for the purpose of strengthening the justification during the publication stage, it would be appreciated if the authors could briefly summarise current evidence regarding the use of omics approaches in septic patients - particularly those in early or emergency department settings. Highlighting

known limitations or gaps in previous studies would help to better define how this study builds on existing knowledge and what novel contributions it aims to offer.

2. The study aims to link multi-omics signatures with clinical outcomes (e.g., mortality, readmission). It would be helpful to briefly describe the analytical strategy for biomarker discovery - e.g., will these markers be validated in independent cohorts, or is this primarily hypothesis-generating?

VERSION 1 - AUTHOR RESPONSE

2. Reviewer Comments to the Authors:

Comments to the Author:
The authors present a well-designed, prospective, multicentre cohort protocol that aims to explore early host immune responses and identify novel biomarkers to improve sepsis diagnosis in the emergency department (ED) setting by the integration of transcriptomics, proteomics, metabolomics, and microbiota profiling. This study is ambitious and aligns well with the goal of advancing precision medicine in sepsis care. The study is methodologically sound and addresses a critical knowledge gap. I would like to commend the team for the thorough planning and comprehensive methodology. Below are a few suggestions to consider for clarity and completeness:

Response: We thank the reviewer for the positive assessment and encouraging comments regarding our study. We also sincerely appreciate the time and effort invested in reviewing our protocol and providing thoughtful suggestions to strengthen the manuscript.

C1. The current version is well written and may not require revision. However, for the purpose of strengthening the justification during the publication stage, it would be appreciated if the authors could briefly summarise current evidence regarding the use of omics approaches in septic patients - particularly those in early or emergency department settings. Highlighting known limitations or gaps in previous studies would help to better define how this study builds on existing knowledge and what novel contributions it aims to offer.

Response: We thank the reviewer for this thoughtful suggestion. In response, we have added a sentence to the introduction highlighting the biological heterogeneity of sepsis and the potential of multi-omics approaches, while noting that such strategies have rarely been applied in early-stage or ED settings. Additionally, we have expanded the discussion to include examples of omics-based studies conducted in the emergency department, including transcriptomic, proteomic, and metabolomic approaches. These studies have shown promising diagnostic and prognostic value but are typically limited to single biological layers and focused on specific outcomes or patient subgroups. To our knowledge, no study has yet applied an integrative multi-omics strategy in a heterogeneous ED population with suspected infection. We hope that the revised text better conveys how our study builds on prior research and may help address gaps in early sepsis recognition.

<u>Addition introduction (page 3, paragraph 2):</u>

'The host response in sepsis is heterogeneous, with distinct biological subgroups identified in ICU cohorts, suggesting that a single biomarker is unlikely to enable accurate diagnosis (Sinha P, Lancet Respir Med, 2023 & Scicluna BP, Lancet Respir Med, 2017). To address this complexity, multi-omics approaches are needed, yet such strategies have rarely been applied in the emergency department, particularly in the early stages of sepsis (Turgman O, Crit Care, 2023).'

<u>Addition discussion (page 11, paragraph 3):</u>
'A number of studies have applied omics-based approaches in emergency department settings, with promising results. For example, the transcriptomic classifiers IMX-BVN-2 and IMX-SEV-2, based on a 29-gene host expression signature, were able to differentiate between bacterial, viral, and non-infectious illness (area under the curve (AUC) 0.80–0.90) and predict in-hospital mortality (AUC 0.84) among ED patients (Galtung N, Eur J Emerg Med, 2022 & Mayhew MB, Nat Commun, 2020). A targeted proteomic study in paediatric ED patients used a three-protein panel (TRAIL, IP-10, and CRP) to distinguish bacterial from viral infections with high accuracy (AUC 0.90–0.92) (van Houten CB, Lancet Infect Dis, 2017). Metabolomic profiling of blood samples from adult ED patients with suspected sepsis identified a six-metabolite panel that distinguished bacteremic sepsis from other acute conditions (AUC 0.93, accuracy 88%) (Kauppi AM, PLoS One, 2016). While these studies support the potential of host-response based diagnostics, they each focus on a single biological layer and typically address pathogen classification or compare highly selected patient groups. To date, no study has applied an integrative omics approach in a heterogeneous ED population of patients with suspected infection to enable early sepsis recognition and biologically meaningful patient stratification.'

C2. The study aims to link multi-omics signatures with clinical outcomes (e.g., mortality, readmission). It would be helpful to briefly describe the analytical strategy for biomarker discovery - e.g., will these markers be validated in independent cohorts, or is this primarily hypothesis-generating?

Response: We appreciate this point. As suggested, we have added a statement to the discussion clarifying that the BIOSEP study is primarily hypothesis-generating. While the study is not designed for external validation, the generated data will inform future biomarker discovery and potential validation in independent cohorts.

<u>Addition discussion (page 12, paragraph 3):</u>
'Given its design, BIOSEP is primarily intended as a hypothesis-generating study. The large-scale integration of multiple omics data will allow for the identification of novel biomarker signatures and immune profiles associated with clinical outcomes. While external validation is beyond the scope of this study, the inclusion of patients from three diverse hospital settings may allow for internal cross-validation or subgroup analyses, enhancing the robustness and generalisability of the findings. These results may inform future validation studies in independent cohorts and guide the development of targeted diagnostic or therapeutic strategies.'
